# Radiographic Indices for Osteoporosis Screening Using CTI and CCR in Resource-Limited Settings: A Literature Review

**DOI:** 10.3390/diagnostics16182914

**Published:** 2026-09-09

**Authors:** Yasaman Gomez-Mazinani, Melissa Ramirez-Villafaña, Eli Efrain Gomez-Ramirez

**Affiliations:** 1 Programa de Servicio Social en Investigación, Facultad de Medicina, Universidad Autónoma de Guadalajara, Zapopan 45129, Mexico; yasaman.gomez@edu.uag.mx; 2 Unidad Académica de Medicina, Universidad Autónoma de Guadalajara, Zapopan 45129, Mexico; melissa.villafana@edu.uag.mx; 3 Departamento de Fisiología, Instituto de Terapéutica Experimental y Clínica (INTEC), Universidad de Guadalajara, Guadalajara 44340, Mexico

**Keywords:** osteoporosis, DXA, radiographic, cortical thickness index, Calcar-to-Canal ratio

## Abstract

Osteoporosis reduces bone integrity, significantly increasing the risk of fractures. This study aims to identify alternative screening methods for assessing osteoporosis in low-income settings, where access to dual-energy X-ray absorptiometry (DXA) is limited. A literature review was conducted, analyzing articles from Google Scholar, PubMed, and institutional libraries published between 2015 and 2025, to identify auxiliary methods for screening for osteoporosis when DXA is not available. Conventional radiographs, when analyzed through the assessment of the Cortical Thickness Index (CTI) and the Calcar-Canal Ratio (CCR), demonstrated high sensitivity and specificity as auxiliary screening tools. However, cutoff values vary significantly among different ethnic populations, indicating the absence of a universal diagnostic standard and underscoring the need for population-specific thresholds. Limited access to DXA worldwide delays early detection of osteoporosis and increases the risk of fragility fractures. Further studies are needed to standardize these metrics in Mexico, which would enable timely diagnosis and treatment in resource-limited settings, thereby improving patient outcomes and quality of life.

## 1. Introduction

Osteoporosis is a chronic skeletal system disease characterized by decreased bone mineral density and the deterioration of bone tissue microarchitecture. This condition reduces bone integrity and mechanical strength, significantly increasing the risk of fractures. In older adults, these fractures affect various areas of quality of life and place a substantial burden on healthcare systems in terms of morbidity, mortality, economic costs, and caregiving needs [1,2,3].

## 2. Materials and Methods

We conducted a systematic review with the aim of identifying alternative screening methods for evaluating osteoporosis in low-income settings where access to dual-energy X-ray absorptiometry (DXA) is limited. We searched studies and national databases such as the Luis Guillermo Ibarra Ibarra National Rehabilitation Institute (INR) of the Mexican government using the PICO framework.

The literature search was conducted between 19 February and 30 March 2026 across searched databases such as PubMed, Google Scholar, and institutional digital libraries. The search strategy were structured around the PICO framework employed Boolean operators such as AND/OR, combining Medical Subject Headings (MeSH) and keywords such as “Osteoporosis,” “Cortical Thickness Index,” “Calcarto-Canal Ratio,” “CTI,” “CCR,” “DXA,” “X-ray,” and “Screening and interpretation of osteoporosis using X-rays” (adapted to the specific syntax of each database).

The review was conducted following a structured search and selection process informed by the Preferred Reporting Items for Systematic Reviews and Meta-Analyses (PRISMA) guidelines. A protocol was developed for the review process; however, it was not prospectively registered.

### 2.1. Selection Criteria

#### 2.1.1. Inclusion Criteria

The inclusion criteria for the documents were as follows: publication year between 2015 and 2025; an older adult population with a diagnosis or suspicion of osteoporosis; evaluation of the utility, accuracy, or feasibility of radiography as a screening tool; evaluation of the Cortical Thickness Index (CTI) and the Calcar-Canal Ratio (CCR); and DXA.

#### 2.1.2. Exclusion Criteria

Articles were excluded if they: did not use X-rays for osteoporosis screening; used screening indices other than CTI or CCR, or if these indices were not collected alongside bone mineral density (BMD); presented associations for predicting fractures in structures other than the femur; employed screening tools other than radiography; addressed osteoporosis associated with comorbidities such as autoimmune, inflammatory, or disabling neuromuscular diseases; were review articles or narrative reviews; or included radiographs with evident fractures or specific disease alterations that could confound the radiographic indices assessment.

### 2.2. Study Selection Process

The literature search was conducted by two independent reviewers. Initially, a total of 199 articles were identified from the following sources: Google Scholar (183), PubMed (12), and institutional databases (4). Subsequently, duplicate documents were manually eliminated by comparing titles, authors, year of publication, journal, and digital object identifier (DOI), when available.

After this screening, the titles and abstracts were reviewed, resulting in a shortlist of 65 documents. These articles were read in full to verify compliance with the inclusion and exclusion criteria. At this stage, 25 articles were excluded for failing to address the main objective or for not meeting the selection criteria, leaving a total of 40 articles.

The remaining 40 documents were evaluated in depth, and a summary was prepared for each one to facilitate their analysis. Following this detailed review, 21 additional articles were excluded: 5 of them because they used radiographic indices other than the CTI or CCR, and 16 because they focused on osteoporosis secondary to systemic comorbidities.

**Figure 1:**
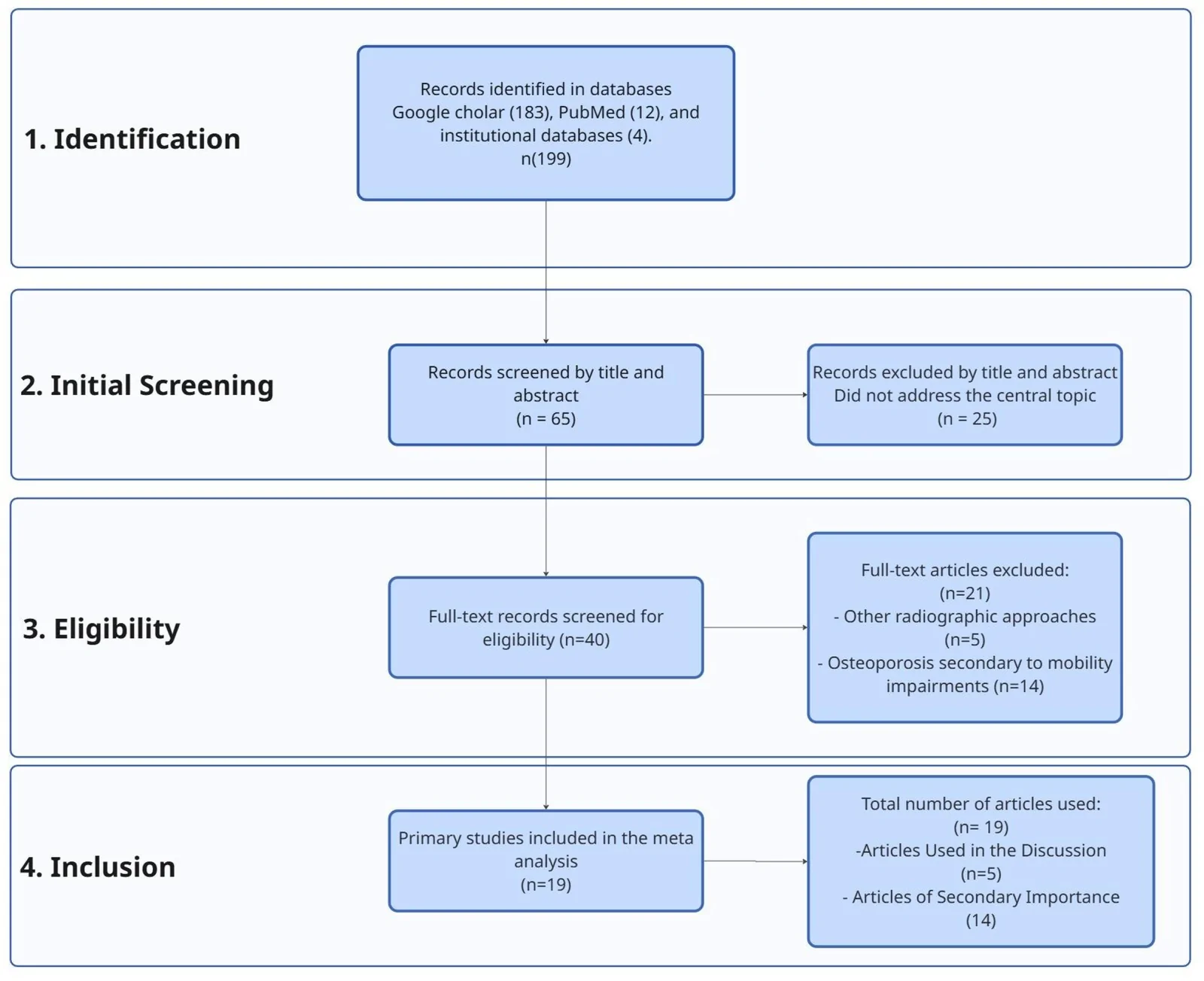
PRISMA diagram.

As a result, the systematic search identified a total of 19 articles that met all eligibility criteria. From this group, 5 articles were selected for the main analysis (Table 1), with the aim of comparing the radiographic indices evaluated and highlighting the utility of radiography as a screening tool. The remaining 14 articles were used to provide background and essential contextual information (Figure 1).

Finally, structured data extraction was performed, organizing the relevant information into a comparative table that included key variables such as year of publication, study population, sample size, and specific.

### 2.3. Use of Artificial Intelligence

Artificial intelligence-based content generation tools, specifically Gemini, were used to facilitate the creation of descriptive images corresponding to CTI. The generated images were used exclusively for illustrative purposes and did not influence the data analysis, the interpretation of results, or the conclusions drawn in the study.

## 3. Background and Evolution of the Concept

Historically, osteoporosis and fractures in old age were considered inherent to normal aging; however, they are currently recognized as manifestations of an age-related disease. Its definition has evolved by incorporating clinical characteristics, BMD, histopathological findings, alterations in bone metabolism, and clinical fracture risk models [9,10,11].

The term was coined in 1829 by Jean Lobstein, who merged the Greek words *osteon* (bone) and *poros* (pores/holes). Clinical understanding advanced significantly in 1941 with Albright, who linked estrogen deficiency to vertebral fractures. In the 1980s, Riggs and colleagues distinguished two types: Type 1 (postmenopausal, associated with low estradiol levels) and Type 2 (senile, related to calcium and vitamin D deficiency). Subsequently, in 1993, the WHO defined osteoporosis as a systemic skeletal disease characterized by low bone mass, microarchitectural deterioration of bone tissue, and a consequent increase in fragility and susceptibility to fractures [9,10,11].

### 3.1. Pathophysiology

Osteoporosis is defined by an imbalance in the bone remodeling cycle, a fundamental metabolic process in adults responsible for repairing micro-damage and contributing to systemic calcium homeostasis. Peak bone mineral density is typically achieved during the third decade of life and may vary depending on genetic, hormonal, and environmental factors [3,12].

1. The Remodeling Cycle and the Basic Multicellular Unit

Under physiological conditions, bone remodeling results from the coordinated activity of osteocytes, osteoblasts, and osteoclasts. The cycle begins when osteoclasts resorb bone matrix, creating a cavity; subsequently, osteoblasts synthesize a new osteoid matrix that will be mineralized. By achieving balance, BMD remains constant [12].

2. Molecular Regulation: The RANKL/RANK/OPG Pathway

Three key elements regulate osteoclastic activity:

RANK: The cell-surface receptor on osteoclasts that activates the final pathway of their development [12,13].

RANKL: A cytokine released by osteoblasts and osteocytes that binds to the RANK receptor on osteoclasts to promote their differentiation and activation [12,13].

Osteoprotegerin (OPG): A decoy receptor secreted by osteoblasts that neutralizes RANKL, thereby inhibiting osteoclast production and activity [12,13].

3. Mechanisms of Bone Loss

The decline in BMD occurs when bone resorption exceeds formation—a phenomenon that typically begins in the third decade.

Estrogen Deficiency: This is the most common cause, particularly after menopause. The lack of estrogen increases RANKL production and decreases OPG expression, thereby enhancing osteoclast recruitment and prolonging osteoclast lifespan, while simultaneously reducing osteoblast survival [12,14].

Aging: Aging is a major determinant of BMD variance. Age-related changes and genetic factors can lead to either a low initial peak BMD or accelerated bone turnover.

Secondary Hyperparathyroidism: Insufficient intake of calcium and vitamin D increases parathyroid hormone (PTH) levels, accelerating bone remodeling and exacerbating the imbalance between formation and resorption [12].

4. Additional Contributing Factors Exacerbating Pathophysiology (Table 2)

Medications: Glucocorticoid therapy is a frequent cause of drug-induced bone loss.

Lifestyle: Physical inactivity (which reduces Wnt signaling pathway activation required for bone formation) and tobacco smoking (which exerts direct toxic effects on osteoblasts) constitute significant risk factors.

Chronic Diseases: Disorders such as diabetes mellitus, hypogonadal states, and malabsorption syndromes also contribute distinctively to the deterioration of bone quality [12].

Risk factors are classified into non-modifiable (intrinsic) and modifiable (extrinsic) [12,15].

### 3.2. Epidemiology and Impact

Osteoporosis is estimated to affect 75 million people across Europe, the United States, and Japan. Globally, the disease affects 200 million women, including approximately one-tenth of women aged 60, one-fifth of women aged 70, two-fifths of women aged 80, and two-thirds of women aged 90. Global demographic projections warn of a significant increase in the number of adults aged 65 and older by 2050. According to these projections, the Mexican population will reach 155,151,000 inhabitants, of whom 35% will be over 50 years old, with an average life expectancy of 80.5 years (5.5 years higher than in 2020). This shifting demographic landscape poses an even greater challenge for healthcare systems, where prevention programs must become a priority [2,16].

During a joint session of the Mexican Academy of Surgery and the National Institute of Rehabilitation “Luis Guillermo Ibarra Ibarra” (INR-LGII) on 30 September 2024, a publication reported that osteoporosis affects 10 million Mexicans, with one in four older adults affected. Furthermore, a fragility fracture occurs every three seconds, and the risk of a second fracture increases by 86%. The convention emphasized the disease’s status as a major public health issue and discussed surgical options for the most common fractures [17].

Bone fragility secondary to osteoporosis becomes clinically evident upon the occurrence of a fracture. In 2019, the World Health Organization recorded 178 million fractures globally, representing a 33.4% increase compared with 1990. Additionally, the absolute prevalence of individuals experiencing acute or chronic symptoms resulting from diagnosed fractures reached 455 million that same year, reflecting a sharp 70.1% increase since 1990. Meanwhile, in Mexico, the Mexican Social Security Institute (IMSS) reported more than 8000 hip fractures in 2019, which constitutes a 42% increase in women and a 40% increase in men compared with 2006. More recent data from 2024 estimate that one in three women and one in five men over the age of 50 will suffer a fragility fracture [2,17,18].

### 3.3. Clinical Manifestations

Osteoporosis predominantly remains asymptomatic until a fracture occurs. In the presence of vertebral fractures, patients may exhibit height loss, back pain, and thoracic kyphosis (which can lead to dyspnea or constipation). Conversely, hip fractures present with pain, swelling, bruising, skeletal deformity, and limited range of motion. Due to resulting functional impairments, fractures of the hip and spine are severe complications associated with high morbidity, mortality, and loss of independence. Upon detection, it is essential to conduct a comprehensive medical history and perform a thorough physical examination to identify underlying etiologies [1,3,12,13].

### 3.4. Diagnosis: DXA and Radiographic Alternatives

The diagnostic capacity for bone health has been radically transformed by technological advances. This refinement process began in 1963 with single-photon absorptiometry (SPA), followed by significant improvements in 1974 with the development of dual-photon absorptiometry (DPA). Finally, in the late 1980s, the introduction of DXA marked a definitive milestone, establishing itself as the gold standard for bone mineral density quantification that prevails to this day [10].

Currently, diagnosis primarily relies on assessing bone mineral density through densitometry or on the presence of a fragility fracture resulting from a fall from standing height or less (low-trauma fractures of the vertebrae, proximal femur, distal radius, humerus, pelvis, proximal tibia, or ribs). An evaluation of asymptomatic fractures can be performed using a spinal X-ray, which frequently correlates with future symptomatic vertebral fractures [3,11,12].

Bone densitometry is a two-dimensional scan that precisely quantifies bone tissue in the total hip, femoral neck, or lumbar spine. It is used both as a diagnostic criterion and as a fundamental predictor of fracture risk; however, it cannot quantify bone depth, which is why individuals of short stature and thin build tend to exhibit lower-than-average BMD. Furthermore, it is considered the best method for determining the rate of bone loss and serves as an indispensable baseline for monitoring and managing disease progression [3,11,12].

Absolute bone density results must be interpreted in relation to patient age, history of fractures, family history, prior medication use, onset of menopause, and other concomitant disorders. DXA results are expressed relative to “normal” values using either a T-score or a Z-score. The WHO defines the T-score as the standard deviation (SD) difference between an individual and a sex-, race-, or ethnicity-matched young adult reference population, representing postmenopausal women and men aged 50 and older. The Z-score should be used to evaluate premenopausal women and men under 50 years old (Table 3) [3,11,12,14,15].

Although DXA is the preferred method for measuring BMD, it is neither routinely requested nor universally available. Developed countries have between 12 and 24 machines per million inhabitants; conversely, developing countries face resource constraints and often have fewer than 1 machine per million inhabitants. Furthermore, in some nations, the lack of reimbursement for DXA scans poses a barrier to identifying and monitoring osteoporosis. The additional cost of the study and the difficulty of accessing it constitute the main disadvantages of this examination, rendering it inaccessible to certain population groups. On the other hand, a direct radiological examination is always obtainable; while it cannot be used as a standalone method for diagnosing osteoporosis or monitoring treatment, it can provide valuable clinical information for physicians [4,6,8].

The imaging method relies on an X-ray tube and an X-ray-sensitive film or image receptor within a cassette, specifically designed to capture the X-ray beam. It was first introduced in the late 1800s, and over time, advancements have been made in image receptors—initially utilizing glass plates, followed by polyester materials, and ultimately digital imaging, which requires less physical space. Currently, the X-ray image is produced when an X-ray beam interacts with specific phosphor crystals contained within intensifying screens inside the film cassette. This process produces light at a specific wavelength that exposes the X-ray film, thereby reducing the required radiation dose [19].

### 3.5. Radiographic Indices in the Hip: CTI and CCR

To this end, both the CTI and the CCR are morphometric indices measured on plain hip radiographs. As fundamental radiographic tools in orthopedics and traumatology, they were originally described by Dorr et al. for selecting appropriate prosthetic designs and evaluating bone quality and morphological changes caused by osteoporosis in the femoral bone prior to hip surgery. Consequently, these indices can serve as essential screening tools when DXA is unavailable [4,7].

The calculation of the CTI on an anteroposterior (AP) hip radiograph is defined as the ratio between the thickness of the bone cortex and the total external diameter of the femur. A standardized procedure is used to quantify this relationship, thereby offering an indirect measure of bone quality and robustness.

The technical methodology for its determination is detailed below:

1. Establishment of the Anatomical Reference Point

The first step involves identifying a key reference point on the radiograph:

- Mid-Lesser Trochanter Line: A horizontal line is drawn through the midpoint of the lesser trochanter. This line is established as the base or “zero” level for all subsequent measurements along the femoral diaphysis.

2. Localization of the Specific Measurement Level

Once the reference line is defined, the exact level where the diameter measurements will be taken is determined:

- 10 cm Distal Level: A distance of 10 cm is measured in a distal (inferior) direction from the mid-lesser trochanter line, following the longitudinal axis of the femur. All diameter measurements must be performed precisely at this level.

3. Measurement of Femoral and Medullary Diameters

At the 10 cm distal level, two fundamental measurements are obtained:

- External Diameter (X): Corresponds to the total diameter of the femoral diaphysis, measured from outer edge to outer edge of the bone at that level.
- Internal Diameter (Y): Represents the diameter of the intramedullary (endosteal) canal—that is, the hollow space in the center of the bone—measured at the same level.

4. Application of the CTI formula

The CTI is defined as the ratio between the thickness of the bone cortex (obtained by subtracting the internal diameter from the external diameter) and the total external diameter of the femur:

$$CTI=\frac{OuterDiameter(X)-InnerDiameter(Y)}{OuterDiameter(X)}$$

In simple terms, this formula could also be represented as $(D_{e}-D_{i})/D_{e}$.

**Figure 2:**
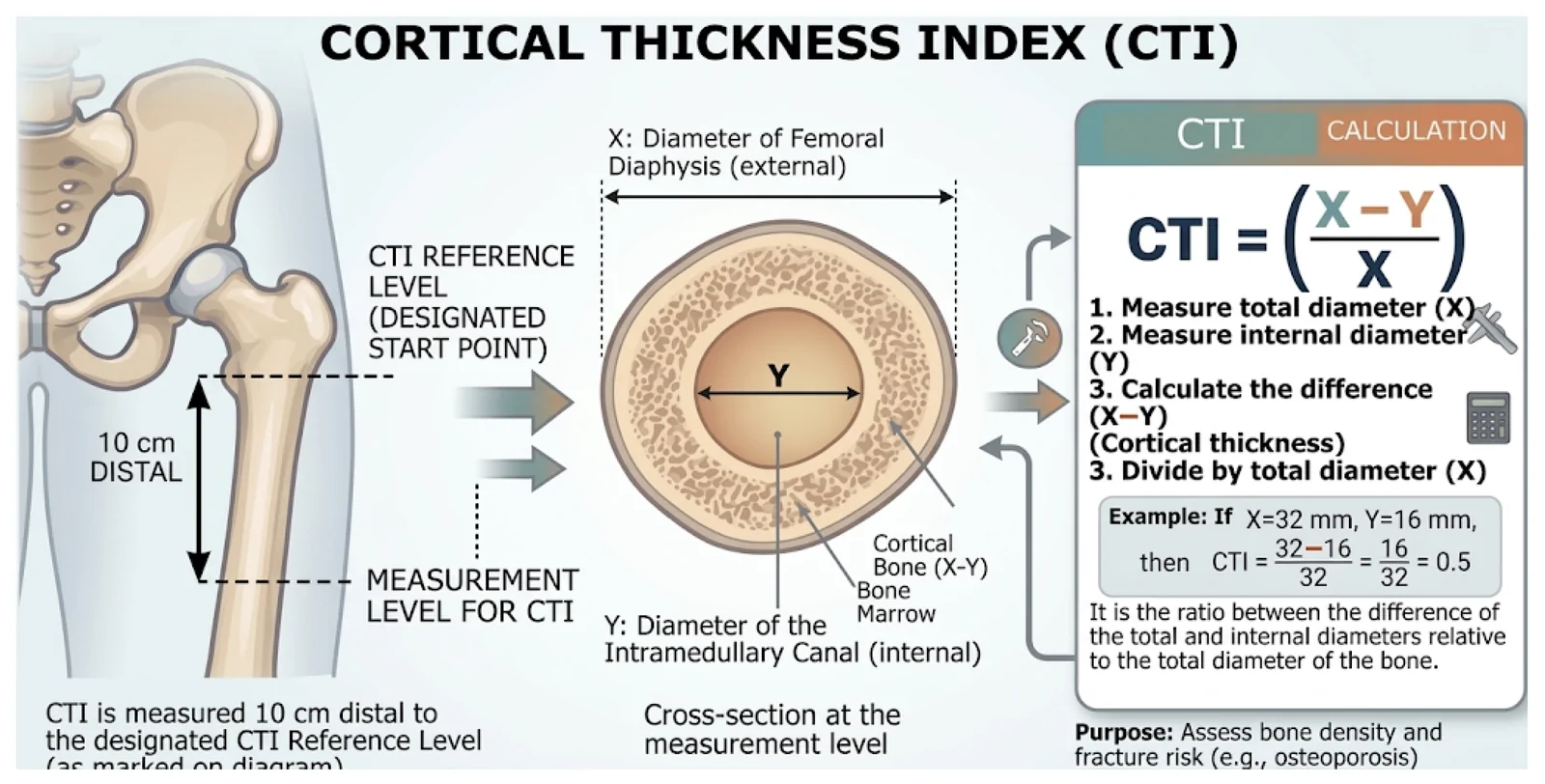
Index measurement CTI. (This schematic is illustrative only, and it should not be interpreted as a validated radiographic protocol). (Google. 2026. Cortical Thickness Index (CTI) Infographic [AI-generated image]. Gemini (Version 3 Flash). https://gemini.google.com/) Translated and edited without AI.

This index provides a valuable quantitative tool for evaluating bone density and cortical robustness, demonstrating utility in the detection and monitoring of bone pathologies, as well as in preoperative planning (Figure 2) [4,5,6,7,8].

The technical methodology for calculating the CCR on an anteroposterior (AP) radiograph of the pelvis or proximal femur is based on specific morphometric measurements of the proximal femur. This process evaluates the relationship between the width of the isthmus canal and the dimension of the calcar canal. To enhance understanding, the procedure is broken down into the following steps:

1. Anatomical Orientation for Proximal Femur Measurements

- Lesser Trochanter Reference: Identify and draw a horizontal reference line passing through the midpoint of the lesser trochanter. This serves as the base level.
- Longitudinal Axis: Establish a reference line along the longitudinal axis of the femur, extending 10 cm inferiorly (distal) from the mid-lesser trochanter line.
- Proximal Endosteal Points: Identify specific points on the internal (endosteal) walls of the medullary canal exactly 3 cm distal to the mid-lesser trochanter line.

2. Internal Diameter Measurements (Excluding Cortical Bone Thickness)

- Calcar Dimension (Variable A): Plot two lines connecting the internal endosteal points at the 3 cm level to the corresponding endosteal points at the 10 cm level along the medial and lateral walls. Project these lines proximally (upward) until they intersect the horizontal mid-lesser trochanter reference line. The internal diameter (the distance between these two projected lines) at this exact baseline level defines the calcar dimension (A).
- Isthmus Diameter (Variable B): This represents the internal diameter of the intramedullary canal measured precisely at level 10 cm distal to the mid-lesser trochanter line.

3. Application of the CCR formula

The CCR is calculated by dividing the internal diameter of the medullary canal at the 10 cm distal level (isthmus) by the internal width of the canal at the level of the calcar (mid-lesser trochanter), using the following formula [4,5,6,7,8]:

$$CCR=\frac{\mathrm{Inner}\mathrm{canal}\mathrm{diameter}10\mathrm{cm}\mathrm{distal}\mathrm{tothe}\mathrm{lesser}\mathrm{trochanter}}{\mathrm{Inner}\mathrm{canal}\mathrm{diameter}\mathrm{at}\mathrm{the}\mathrm{lesser}\mathrm{trochanter}}$$

## 4. Results and Discussion

Osteoporosis is a chronic disease of the skeletal system that has been studied over the years, leading to advances in our understanding and the development of tools to help identify it. Currently, bone densitometry is recognized as the gold standard. In developing countries, however, availability is limited, shortages are common, and economic constraints persist due to the additional cost of the test. This lack of access hinders timely diagnosis and the initiation of preventive treatment, leading to complications such as fragility fractures, which increase morbidity, mortality, and the economic burden.

This is a public health issue—compounded by an increasingly aging population—and efforts are underway to find tools to address this gap so that the population can meet health goals regardless of income or location, receiving equitable, high-quality care.

To this end, screening methods can be used to detect this disease; the use of CTI and CCR is proposed as an indirect morphometric tool for identifying bone structural changes through X-rays, rather than directly measuring BMD or replacing DXA. Their potential integration into primary care settings represents a promising hypothesis for opportunistic screening that requires further clinical validation.

Although information is limited, several articles are presented that can help guide their use. Five articles were analyzed: Köse et al. [4], a prospective study; Nguyen et al. [5]; Burli et al. [6], a cross-sectional study; Ilyas & Ipci [7], a cross-sectional descriptive study; and Wiguna et al. [8], a cross-sectional study using a correlation test design. All studies included a comparison with DXA and RX. The total study population consisted of older adults ranging in age from 63 to 73 years, with participants ranging from 50 years to the maximum reported age of 93 years. Although the sample sizes were not small, they used various cutoff values [4].

Nguyen et al. [5] stands out for its largest cohort—560 screened volunteers and 424 final candidates—and is unique in that it includes both genders: 260 women and 164 men (a 61.32% to 38.68% ratio). Given biological differences in bone geometry and strength, this allows for the identification of cutoff values. On the other hand, Burli et al. [6] present a mixed study sample of 80 patients, 53 women and 27 men; of those with osteoporosis, 28 were women, and 8 were men (35% and 10% of the total study population). The remaining articles in the study population are Ilyas & Ipci [7] with 156 patients, Köse et al. [4] with 92 patients, and Wiguna et al. [8], which had 34 subjects—17 with osteoporosis (50%) and 17 controls. Burli et al. [6] included patients who had hip fractures, and this fracture may have altered bone density at the femoral neck.

The eligibility criterion for patient selection was age. Patients with a history of previous hip surgery, femoral or vertebral fractures, a history of proximal femoral malformations or metabolic bone disorders such as Paget’s disease, steroid use, or treatment with bisphosphonates for more than one year were excluded. A perceived bias in the articles by Nguyen et al. [5] and Ilyas & Ipci [7] included a population with osteoarthritis, which may have led to elevated results in this study population due to the described inverse relationship between osteoporosis and osteoarthritis.

Köse et al. [4] used a translucent ruler and a pencil for some measurements, while the rest were performed with software. It was found that the ROC curve was used to determine threshold values, sensitivity, and specificity in the articles by Köse et al. [4], Ilyas & Ipci [7], and Wiguna et al. [8]. The Landis and Koch criteria were used to classify and quantify the level of reliability in Köse et al. [4], Nguyen et al. [5], and Burli et al. [6]. Spearman’s criteria were used to determine the strength and direction of the relationship in Nguyen et al. [5], Ilyas & Ipci [7], and Wiguna et al. [8]. In addition, Nguyen et al. [5] conducted a binary logistic regression analysis to assess CTI capacity, and Burli et al. [6] evaluated the predisposition to osteoporosis by sex using the chi-square test (which demonstrated that women are more likely to develop osteoporosis).

The cutoff values presented vary depending on the study, with this variation resulting from genetic traits, dietary habits, and geographic differences. Köse et al. [4] in Turkey found greater variations by ethnicity; they evaluated the CTI with a threshold value of less than 0.3 and the CCR at 0.47 for the Turkish population (detecting the presence of osteoporosis with 100% sensitivity and 98% specificity both index); with bone densitometry values (*p* = 0.001), a BMD index CTI 0.57 indicates osteoporosis in the Korean population, and a CCR index below 0.57 indicates osteoporosis in the Chinese population. Nguyen et al. [5] reported CTI values for men of 0.62 (with a sensitivity of 57.7% and a specificity of 69.8%) and for women of 0.56 (with a sensitivity of 92.8% and a specificity of 24.2%), with *p* < 0.001. Burli et al. [6] studied an Indian population, with a CTI cutoff of 0.43 (100%, specificity was 74.58%) and a correlation with DXA of r = 0.8172, *p* < 0.0001, and a CCR of 0.50 (sensitivity was 100%, specificity was 75.86%), compared to DXA with r = 0.8188, *p* < 0.0001. Ilyas & Ipci [7], Turkey, reported a cutoff of CTI 50.4 degrees (sensitivity: 83% and specificity: 83%) and CCR of 60.3 degrees (sensitivity: 78, specificity: 77%). Wiguna et al. [8] reported a CTI of <0.57 with 70.59% sensitivity and 76.47% specificity (Table 4).

The reviewed evidence suggests that the CTI and the CCR, obtained via conventional hip radiography, are viable tools. However, several methodological limitations of this systematic review must be acknowledged. First, there was substantial clinical and methodological heterogeneity among the included studies, driven by wide variations in participant age ranges and the inclusion of specific clinical subgroups (such as patients with osteoarthritis or acute hip fractures) that inherently alter local cortical anatomy. Second, we observed significant inconsistency across the literature regarding measurement techniques and reported cut-off values, limiting direct comparability. Third, a quantitative meta-analysis could not be performed due to data reporting gaps in primary studies, specifically the omission of standard deviations and 95% confidence intervals. Finally, ethnic variability in cut-off points represents a major limitation; this international heterogeneity highlights the lack of a universal standard and underscores the complete absence of external validation in the target population, emphasizing the need for local research to define thresholds tailored specifically to the Mexican population.

In this regard, incorporating CTI and CCR into primary care could be a cost-effective strategy in settings with limited access to DXA. Defining national normative parameters would promote early detection, prevent fragility fractures, and reduce the economic impact and the morbidity and mortality associated with osteoporosis in Mexico.

## 5. Conclusions

Although dual-energy X-ray absorptiometry remains the gold standard for diagnosing osteoporosis, its availability is limited in various global contexts. This lack of access hinders timely diagnosis and early-stage management, leaving patients vulnerable to severe complications such as fragility fractures, which increase morbidity and mortality while imposing a substantial economic burden. Given this scenario, exploring auxiliary screening methods is essential. Although the application of conventional radiography in this field is not a novel line of research, a gap in evidence remains to fully validate its clinical scope. The data extracted from these articles demonstrate high sensitivity and specificity for the Cortical Thickness Index and Calcar-Canal Ratio, supported by robust methodology, ROC curves, and Landis-Koch plots, suggesting their utility as auxiliary risk-screening tools when bone densitometry is unavailable.

However, the absence of population-specific cut-off points for Cortical Thickness Index and Calcar-Canal Ratio measurements currently limits their active implementation in Mexican clinical practice. These findings underscore the need for further studies aimed at standardizing these metrics as auxiliary diagnostic tools. Establishing these thresholds could enhance early diagnostic and therapeutic approaches in settings across Mexico where DXA is inaccessible, ultimately improving patient quality of life.

## Figures and Tables

**Table 1:** Included studies.

Study	Country	Population	n	Age
Köse et al. (2015) [4]	Turkish	Older Adult	Women (n = 92)	Age range, 60–93
Nguyen et al. (2018) [5]	Japanese	Population with Osteoarthritis Old adult	Men (n = 164) Women (n = 260)	Men age range 50–90 Women age range 52–93
Burli et al. (2020) [6]	Indian	Older Adult	Women (n = 28) Men (n = 8)	Age range 50–89
Ilyas & Ipci (2023) [7]	Turkish	Older Adult	Women (n = 156)	Age range 68.27 ± 8.27
Wiguna et al. (2024) [8]	Indonesian	Older Adult	Woman (n = 17)	Age range over 65 years

**Table 2:** Major risk factors and mechanisms of bone loss.

Intrinsic Factors (Non-Modifiable)	Extrinsic Factors (Modifiable)
**Genetics:** Determines 50% to 80% of peak bone mass variance.	**Nutrition:** Calcium intake <1200 mg/day or Vitamin D deficiency (<30 ng/mL). Malabsorption, low sun exposure, excessive protein intake in unbalanced diets, excessive phosphate intake, or high sodium intake that increases urinary calcium excretion.
**Age:** Physiological decline in bone density after the fourth decade of life.	**Toxic & Lifestyle Habits:** Tobacco smoking and excessive alcohol consumption (exerting direct toxicity on osteoblasts), and sedentary lifestyle
**Sex and Ethnicity:** Higher prevalence in Caucasian and Asian women.	**Endocrine Factors:** Late menarche or menstrual cycle alterations (conditions associated with low bone mass); surgical or non-surgical menopause before age 45; hormonal infertility; and premenopausal estrogen deficiency resulting from anovulation due to anorexia nervosa, excessive exercise, or psychological stress.
**Congenital Disorders:** Osteogenesis imperfecta, Turner syndrome, and Marfan syndrome.	**Iatrogenic Factors:** Prolonged use of glucocorticoids, proton pump inhibitors (PPIs), anticonvulsants, or excessive thyroid hormone replacement therapy.

**Table 3:** Diagnosis of osteoporosis.

Diagnosis	T-Score Range (SD)
**Normal**	−1 above
**Osteopenia** *(Low Bone Mineral Density)*	Between −1 and −2.5
**Osteoporosis**	−2.5 or lower (≤−2.5)
**Severe Osteoporosis**	−2.5 or lower (≤−2.5) + confirmed fracture

**Table 4:** Diagnostic Comparison of Radiological Indices.

Author, Year, and Country	Population	Index Cut	Parameter	Sensitivity	Specificity	Results
Köse et al. [4] Turkish	Older Adults (Age range 72.0 ± 8.9 years) Women (n = 92)	0.3	CTI	100% CI 95%	98%	Correlation with BMD: r: 0.591, *p* < 0.001
Köse et al. [4] Turkish	Older Adult (Age range, 60–93 years) Women (n = 92)	0.47	CCR	100%	98%	Correlation with BMD: r: −0.303, *p*: 0.003
Nguyen et al. [5] Japanese	Population with Osteoarthritis Men (n = 164) (Age range 50–90 years)	0.62	CTI	57.7%	69.8%	Correlation with BMD: r: 0.40, *p* < 0.001
Nguyen et al. [5] Japanese	Population with Osteoarthritis: Women (n = 260) Older Adult (Age range 52–93 years)	0.56	CTI	92.8%	24.2%	Correlation with BMD: r: 0.52, *p* < 0.001
Burli et al. [6] Indian	Women (n = 28) Men (n = 8) Older Adult (Age range 50–89 years)	0.43	CTI (AP)	100% No CI 95%	74.58%	Correlation with BMD T-score: r: 0.82, *p* < 0.001
Burli et al. [6] Indian	Women (n = 28) Men (n = 8) Older Adult (Age range 50–89 years)	0.50	CCR	100% No CI 95%	75.86%	Correlation with BMD T-score: r: 0.818, *p* < 0.001
Ilyas & Ipci [7] Turkish	Women (n = 156) Older Adult (Age range 68.27 ± 8.27 years) Population with Osteoarthritis	50.4 degrees	CTI	83%	83%	Correlation T-score: Total hip: r: 0.632, *p* < 0.001 Femoral neck r: 0.513, *p* < 0.001
Ilyas & Ipci [7] Turkish	Women (n = 156) Older Adult (Age range 68.27 ± 8.27 years) Population with Osteoarthritis	60.3 degrees	CCR	78%	77%	Correlation T-score: Total hip: r: −0.495, *p* < 0.001 Femoral neck r: −0.445, *p* < 0.001
Wiguna et al. [8] Indonesian (Bali)	Woman (n = 17) Older Adult (Age range over 65 years)	<0.57	CTI	70.59%	76.47%	Correlation with BMD r: −0.387, *p* < 0.024

## Data Availability

No new data were created or analyzed in this study. Data sharing is not applicable to this article.
